# Rapid dataset generation methods for stacked construction solid waste based on machine vision and deep learning

**DOI:** 10.1371/journal.pone.0296666

**Published:** 2024-01-16

**Authors:** Tianchen Ji, Jiantao Li, Huaiying Fang, RenCheng Zhang, Jianhong Yang, Lulu Fan

**Affiliations:** 1 College of Mechanical Engineering and Automation, Huaqiao University, Xiamen, Fujian, China; 2 Shenzhen Municipal Engineering Corporation, Shenzhen, Guangdong, China; Chitkara University, INDIA

## Abstract

The development of urbanization has brought convenience to people, but it has also brought a lot of harmful construction solid waste. The machine vision detection algorithm is the crucial technology for finely sorting solid waste, which is faster and more stable than traditional methods. However, accurate identification relies on large datasets, while the datasets from the field working conditions are scarce, and the manual annotation cost of datasets is high. To rapidly and automatically generate datasets for stacked construction waste, an acquisition and detection platform was built to automatically collect different groups of RGB-D images for instances labeling. Then, based on the distribution points generation theory and data augmentation algorithm, a rapid-generation method for synthetic construction solid waste datasets was proposed. Additionally, two automatic annotation methods for real stacked construction solid waste datasets based on semi-supervised self-training and RGB-D fusion edge detection were proposed, and datasets under real-world conditions yield better models training results. Finally, two different working conditions were designed to validate these methods. Under the simple working condition, the generated dataset achieved an F1-score of 95.98, higher than 94.81 for the manually labeled dataset. In the complicated working condition, the F1-score obtained by the rapid generation method reached 97.74. In contrast, the F1-score of the dataset obtained manually labeled was only 85.97, which demonstrates the effectiveness of proposed approaches.

## Introduction

With the continuous development of urbanization, annual solid waste is also increasing. In China, the construction solid waste generated yearly is as high as 2.4 billion tons, but only 10% is properly disposed of [1]. A large amount of solid waste pollutes the environment, occupies land resources, and causes disasters such as landslides [2, 3]. The classification, recycling and reuse of solid waste can effectively save resources and reduce environmental pollution, with waste classification serving as the initial crucial phase. Some conventional sorting methods can achieve preliminary classification, but their discriminative ability is limited, resulting in low levels of classification and value for recyclables. On the other hand, detection-based waste sorting (photoelectric sorting and machine vision sorting) enables high-value recycling, with its detection algorithms being the key technology for precise sorting [4]. Among them, machine vision detection based on deep learning has been widely studied due to its advantages of fast speed, high accuracy, low cost, and strong adaptability [5–8]. Our previous research has also demonstrated that the instance segmentation algorithm not only categorizes waste, but also accurately segments object contours, enabling precise grasping by the robot [9].

However, the accuracy and robustness of the deep learning based detection depend on a large dataset that meets the field working conditions. Although neural networks have been widely used in solid waste prediction, obtaining datasets consistent with the field working conditions takes time and effort [10]. In addition, instance segmentation datasets used for waste detection require the annotation of the contours and types of each object in the images. To ensure correctness, the annotation of instance segmentation datasets still relies mainly on time-consuming manual labeling [11]. And the irregular shapes and stacking conditions of solid waste increase the workload and error rate.

The concept of synthetic dataset has been proven to be a more convenient and efficient method for data annotation [12]. It primarily includes the synthesis of new datasets using existing data and the generation of realistic datasets through the construction of virtual data. Among the various methodologies, copy-paste is a more effective way to augment instance segmentation datasets. Based on the bounding box (bbox) of the RGB-D dataset of small household items, Fully Convolutional Network (FCN) was used to infer the mask information of the instance and randomly paste the obtained instance to the background of the kitchen [13]. Another study [14] focused on contextual information under the same working condition and pasted household items on the position detected as desktops and other platforms. Both pieces of research demonstrated the effectiveness of copy-paste and hybrid training on generated and real datasets. Some studies focus on the importance of contextual information, such as Dvornik et al. and Fang et al. using the context network [15] and contextual correlation heat map algorithm [16], respectively, to calculate the correlation between the instance and the background in order to paste the object in a more appropriate place, which were proven to be effective. But Google Team’s research has proved that modeling without using context information can also achieve better results [17]. It also proved that iterative self-training combined with copy-paste can effectively improve the detection accuracy.

For dataset instance segmentation, recent studies are mainly based on traditional image processing and deep learning. Some of them [18–20] proposed to map the 3D modeling of workers and construction equipment onto different 2D backgrounds (authentic images), simulating various working conditions and quickly obtain segmentation information through the relationship between 3D models and the backgrounds. Such methods can create complex and diverse working conditions, but challenging to imitate construction waste with various shapes and appearances. There are also methods based on edge detection, using operators such as Canny [21], Laplacian of Gaussian (LoG) [22] to detect contour edges, but few related studies on instance segmentation were conducted. For deep learning, DeepCut [23] achieved the effect of obtaining instance segmentation results given weakly supervised bounding boxes. The weakly supervised model can also generate pixel point annotations through image-level tags and bounding boxes [24].

However, most of the copy-paste methods and deep learning based automatic labeling methods rely on weakly supervised annotations or labeled datasets. While traditional image-based instance segmentation methods are weak in category recognition and contour detection, it is challenging to achieve good segmentation results using only unimodal information. As a result, when the target object or the waste sorting condition changes, it is impossible to quickly generate a dataset or detection model under the new condition. Moreover, there are materials with irregular shapes and sizes in the actual sorting condition, and complex conditions such as adhesion and stacking often occur. In this case, the effect of instance segmentation affects the accuracy of the final mechanical sorting and can guide the order of grabbing stacked objects [25]. However, few studies show how the dataset simulates complex and changeable operating conditions, or how to automatically annotate complex stacked datasets with minimal manual labor.

This paper aims to realize the rapid generation of solid waste datasets under different working environments and stacked scenarios without relying on manual labeling. An acquisition and detection platform was built using matched dual cameras for RGB-D image acquisition. A dataset rapid generation method was proposed, which can produce trainable dataset without manual annotation. Two automated labeling methods were proposed for real-world stacked datasets, enabling rapid annotation of complex data without manual labeling. Finally, two working conditions: the simple working condition and the complicated working condition, were designed to verify the method’s effectiveness. The detection experiments of two working conditions were designed to verify the methods’ validity.

## Material and methods

**Fig 1:**
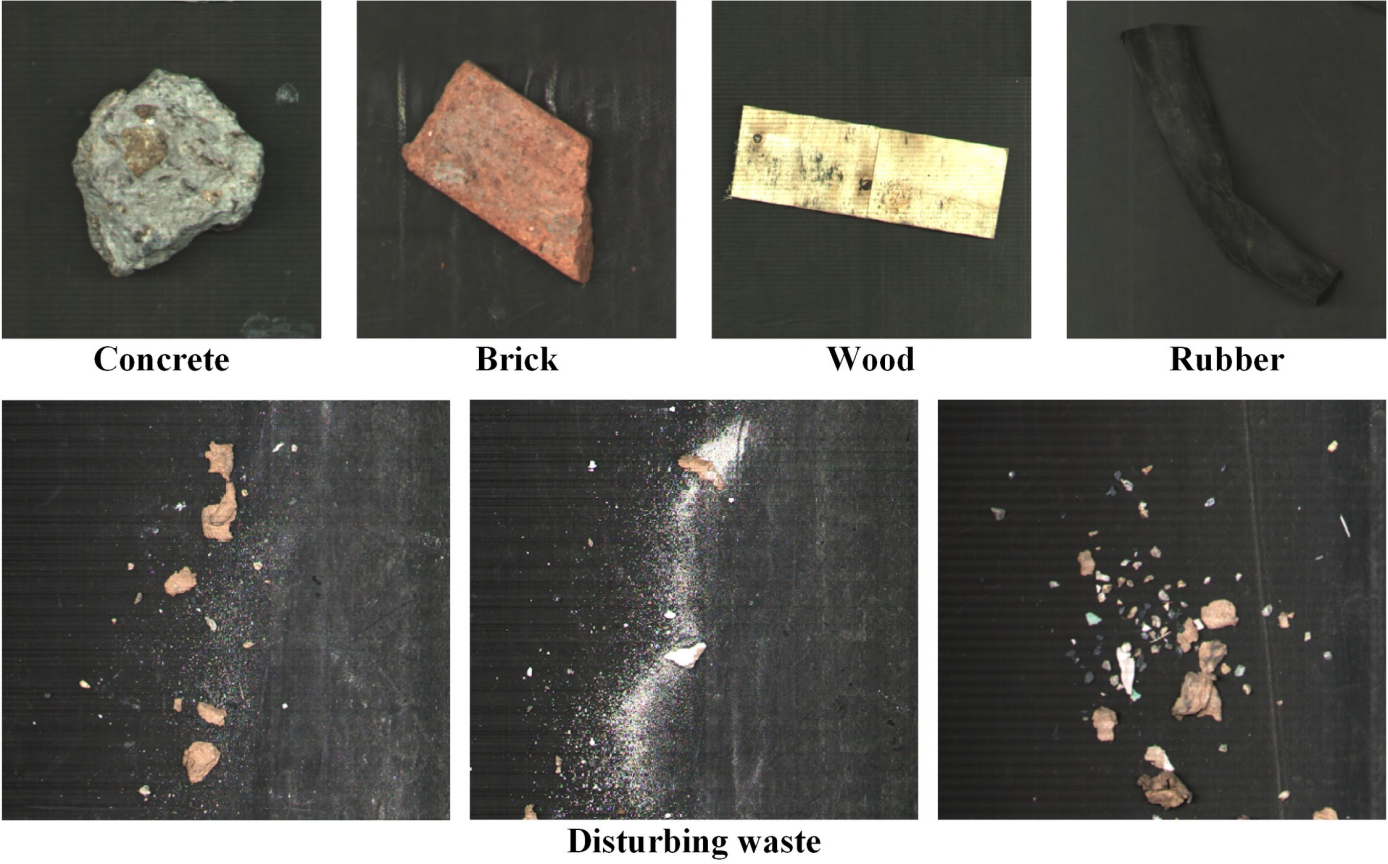
Construction solid waste samples and disturbing waste.

The main purpose of this research is to quickly and automatically annotate construction waste datasets. Therefore, 298 common construction solid waste samples were obtained for experimentation, including four categories: 95 bricks, 60 concrete, 79 wood, and 64 rubber (**Fig 1**). Due to the difficulty in acquiring the samples, all available samples were included in the experiment, resulting in some imbalance in sample quantity. Additionally, this section introduces an acquisition and detection experimental platform constructed by the team and a method based on the distribution points generation theory and data augmentation algorithm. In order to quickly obtain annotated real-world data to augment the dataset, two automatic annotation methods based on semi-supervised self-training and RGB-D fusion edge detection were proposed.

### Material

The construction solid waste used in the experiment was collected locally in Quanzhou City, Fujian Province. Four kinds of typical construction solid waste (as shown in **Fig 1**) were selected as experimental materials: concrete, brick, rubber, and wood.

To approximate the field construction solid waste sorting constructions, waste paper, and gravel were selected as the interference objects to generate background images (**Fig 1**) under complicated conditions.

### Acquisition and detection platform

**Fig 2:**
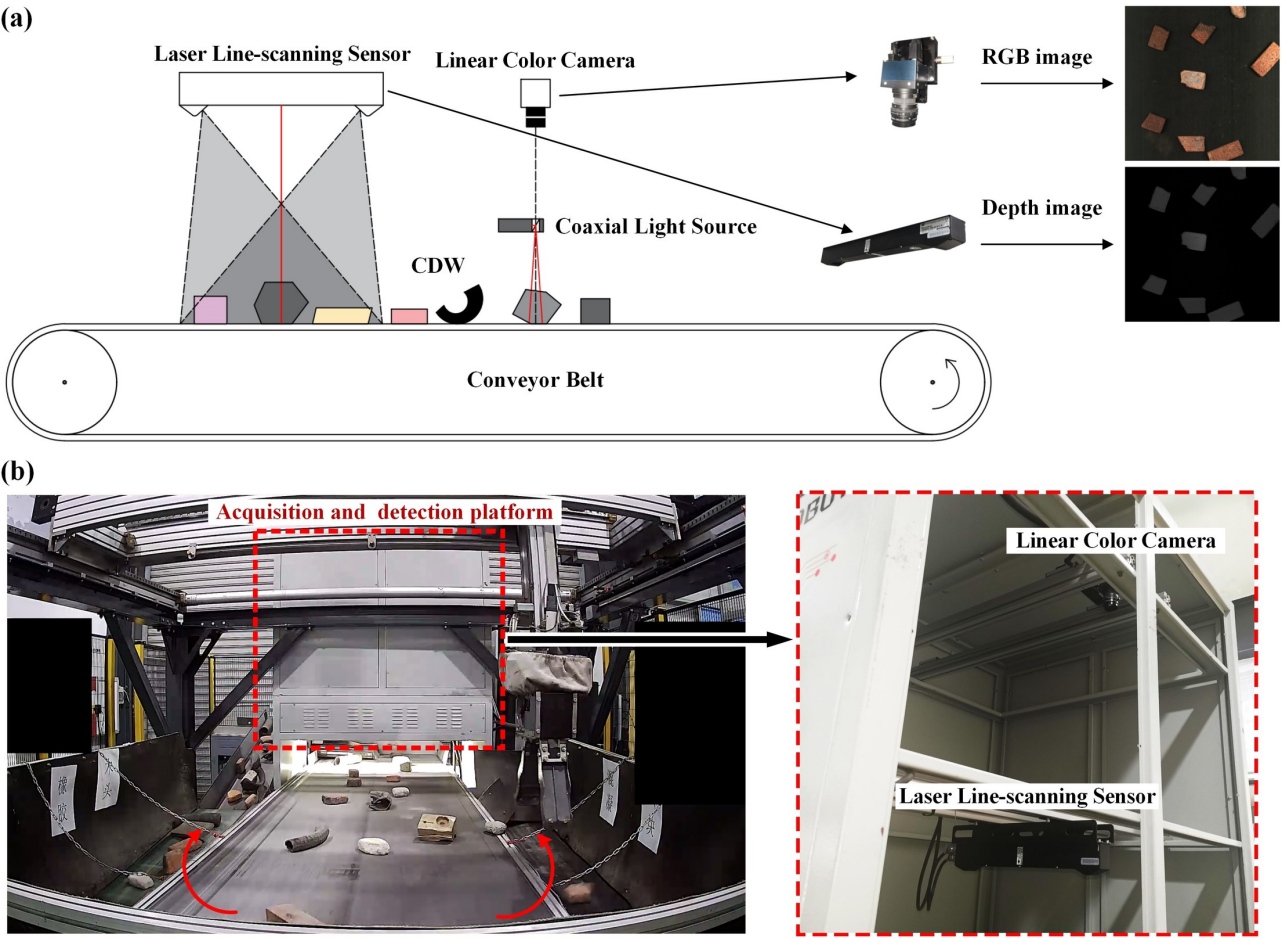
Construction solid waste acquisition and detection platform: (a) the schematic diagram of the acquisition and detection platform, (b) the actual picture of the acquisition and detection platform.

To ensure the acquisition of high-quality images and enable rapid annotation, an acquisition and detection platform was built on a black conveyor belt for the collection of RGB and depth images. As shown in **Fig 2,** the platform includes RGB and depth imaging modules. The RGB imaging module comprises a linear color camera (DALSA 4K GigE Vision™, America) and coaxial light source (OPT, China), while the height imaging module contains a laser line-scanning sensor (LMI Gocator 2880, Canada) to collect depth images of objects. In order to align the collected RGB images with the depth images in space, an encoder was used to convert the displacement of the conveyor belt into pulse signals in a fixed proportion and then triggered two modules to collect images simultaneously. To fully leverage the limited quantity of waste and the information from different perspectives, a cyclic conveyor belt was employed to elevate the material and allow it to redistribute on the conveyor belt. To control the quantity of collected data, a fixed amount of time is allocated for each collection. Finally, empty images were removed from the dataset, resulting in a slight discrepancy between the number of original images and objects.

It should be noted that the experimental equipment and data are owned by the author team, so no authorization is required.

### Automatic annotation of instances

**Fig 3:**
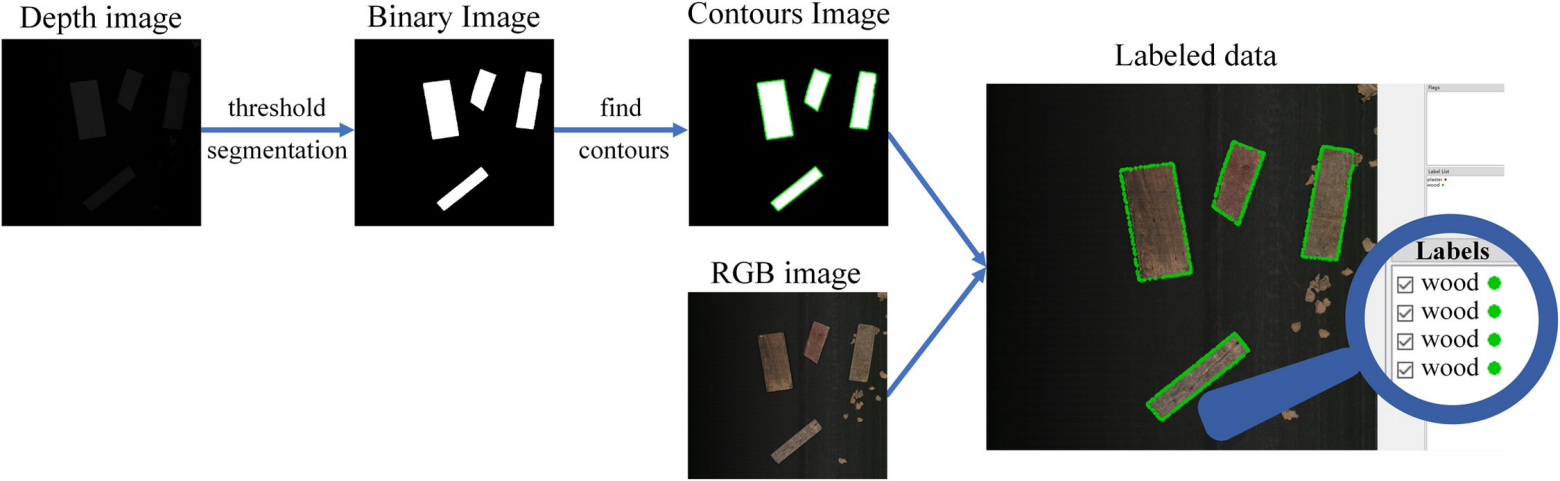
Labeled data are automatically generated from the depth and RGB images.

This research proposes a simple method to generate annotation automatically without manual labeling. The flowchart of this method is shown in **Fig 3**, and the specific steps are as follows:

1. **RGB and depth images:** The images were collected in different batches. When collecting images of each batch, the objects on the conveyor belt are of the same category and sparsely placed. The collected results are shown in **Fig 3**. RGB images can display the color and texture information of the objects, and depth images can display the contour and height information of the objects.
2. **Contour images:** The conveyor belt may present unrelated interferences in addition to the objects that need to be classified. The height of unrelated interferences is significantly smaller than the heights of objects to be classified. Therefore, the depth information can be used to remove the unrelated interferences on the conveyor belt to obtain objects’ precise contours. As shown in **Eq (1)**, the thresh is set to a certain depth value (20), the maxval is 255, and the resulting binary images are shown in **Fig 3**. Then, the contour search method based on topological structural analysis [26] is used to find the contours of the objects and obtain the instances.
$$dst(x,y)=\left\{\begin{array}{cc} \;max\;val & \;if\;src(x,y)>\;thresh\\ 0 & \;otherwise \end{array}\right.$$ (1)
3. **Labeled data:** When capturing contour images, it was equivalent to obtaining the contour label of objects. Since the objects in the images are all of the same category, manual classification of each individual object is unnecessary. Then the collected RGB-D images can be used to get the training set. The Labeled data in **Fig 3** is the display result of the dataset on the Labelme software.

### Stacked dataset rapid generation method

In the previous section, a method for automated annotation generation is presented. In order to ensure the accuracy of the labeled contours, objects are intentionally arranged sparsely, and all objects in the same group belong to the same category. However, in the field working condition (i.e., the real scenario), all objects in the images are of different types, and these objects will adhere to each other. The produced dataset has limitations and cannot be applied to natural working conditions. And in this section, a rapid generation method was proposed to augment the automatically annotated instances.

**Fig 4:**
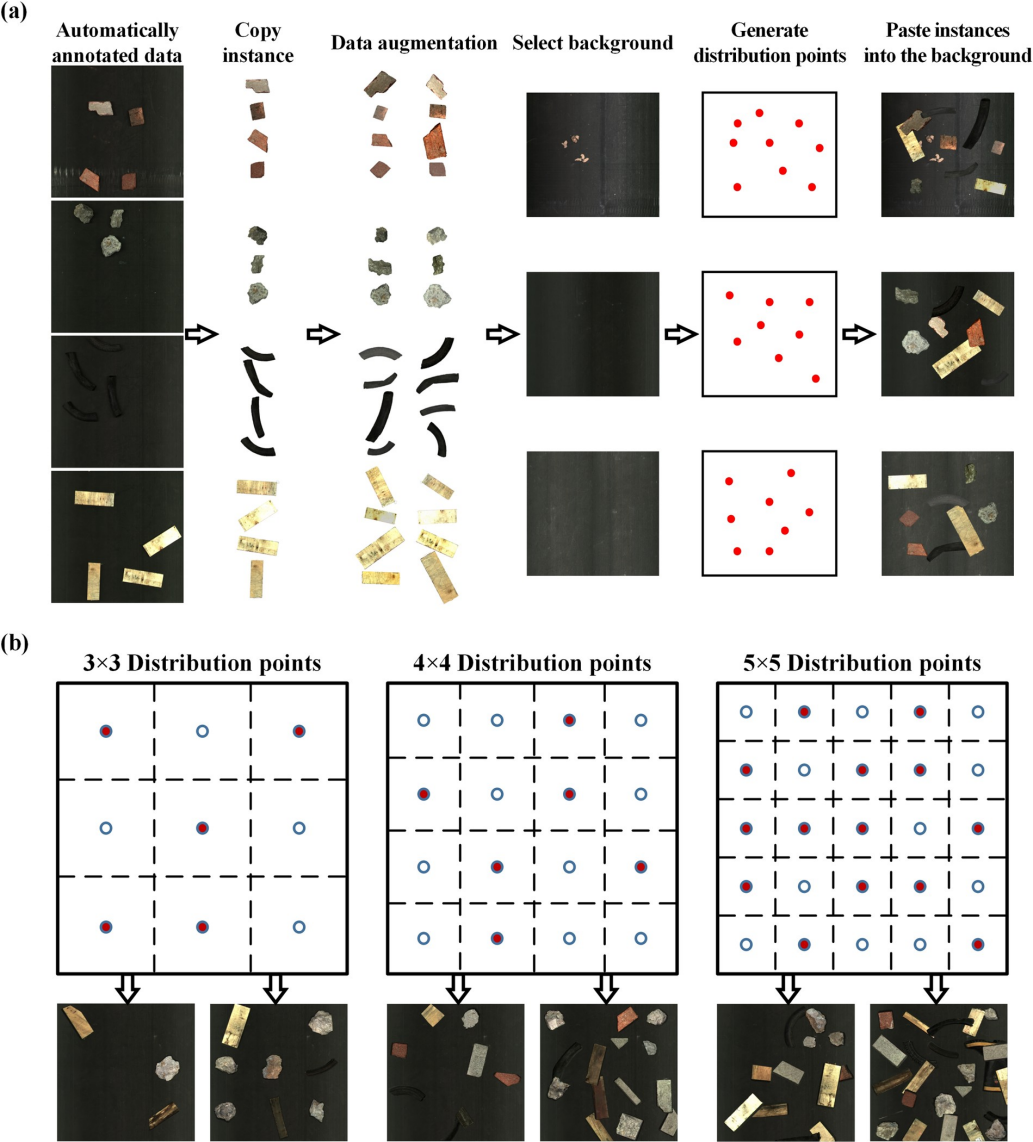
The theory and details of the rapid generation method: (a) the proposed copy-paste process, (b) generate distribution points.

The main steps of the approach are shown in **Fig 4(A)** and described below. It primarily consists of four steps: **1)** Acquiring waste instances and performing data augmentation; **2)** Capturing the background images; **3)** Generating distribution points to determine the positions where instances will be pasted; **4)** Pasting the instances onto the background images to generate a rapid-generation dataset.

**Fig 5:**
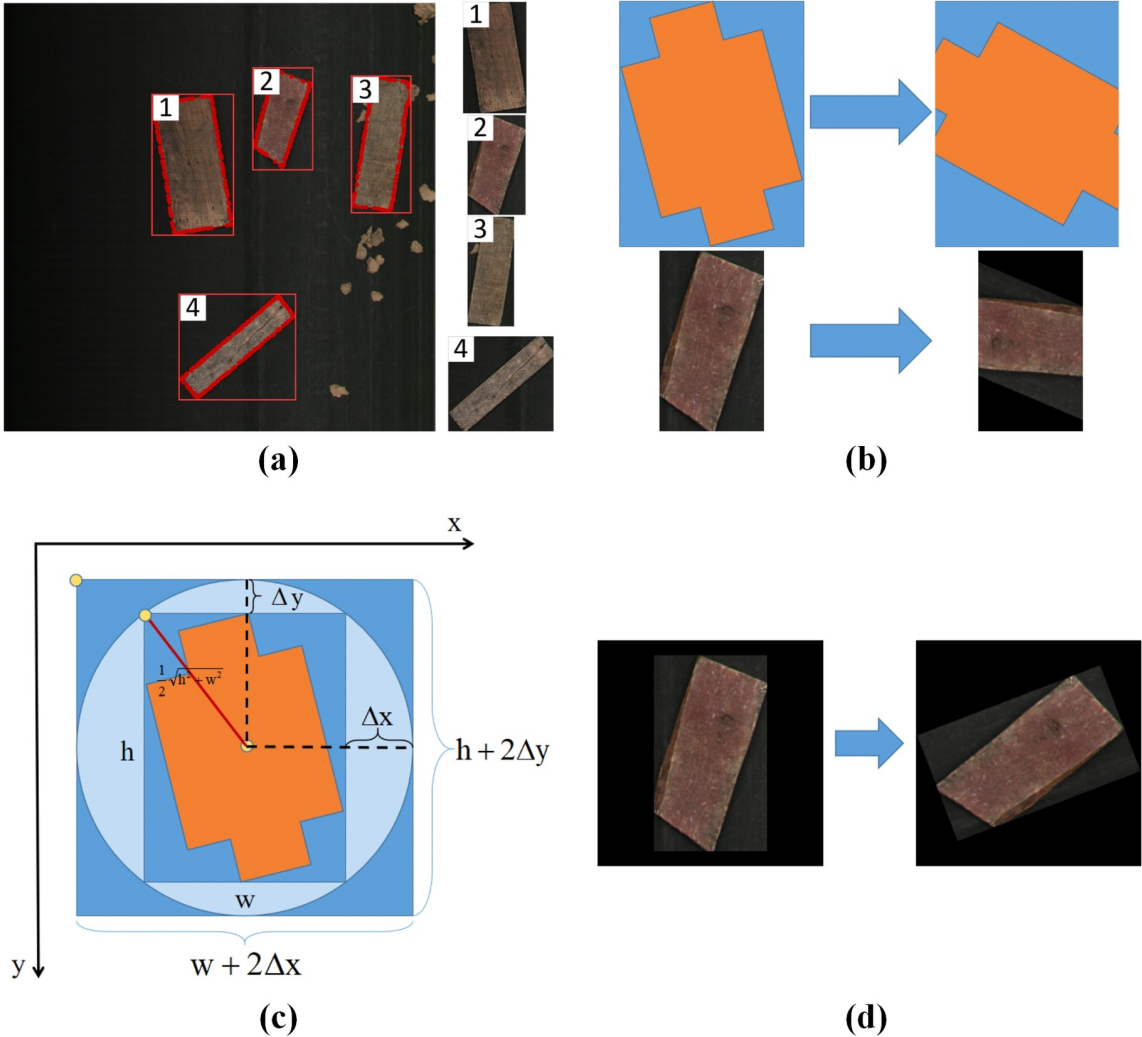
Details about copying and enhancing each instance: (a) cutting off the objects from the image, (b) the loss of edge information due to rotation, (c) the mathematical modeling of the object image, (d) the rotation of an object after expanding image edges.

**1) Instance acquisition and augmentation**
  According to the annotated dataset, the contour point set surrounding the object can be obtained, and the peripheral rectangle of the object can be obtained according to the contour point set. The object can be cut off from the original picture with the peripheral rectangle, as shown in **Fig 5(A)**.

After cutting off the objects, data augmentation is required to increase their diversity. In this article, horizontal and vertical flipping, image rotation, and standard lighting changes were used to simulate different environmental conditions where instances were randomly scattered on the conveyor belt. The idea is to manipulate the spatial position, brightness, and other attributes of the objects to make them closer to real working conditions, so as to improve the accuracy of the detection algorithm. However, it should be noted that common rotation transformations cannot be directly carried applied to the instances, as this may lead to the loss of edge information and result in specific adverse effects (**Fig 5(B)**). Therefore, a specially designed instance rotation method was purposed.

**Firstly,** the edge of the object was extended. Then, the instance was rotated according to the mathematical modeling of the object image (**Fig 5(C)**). The radius *r* of the image’s peripheral circle can be obtained according to **Eq (2)**, where *h* and *w* are the height and width of the object image.


$$r=\frac{1}{2}\sqrt{h^{2}+w^{2}}$$ (2)

According to **Eqs (3)** and **(4)**, the image edge’s extended lengths Δ*x* and Δ*y* can be obtained, respectively. After edge expansion, the height of the object image is *h*+2Δ*y*, and the width is *w*+2Δ*x*.


$$\Delta x=r-\frac{1}{2}w$$ (3)

$$\Delta y=r-\frac{1}{2}h$$ (4)

**Fig 5(D)** shows the object image after edge expansion, and the expanded part is the black area with a grayscale of 0. After edge expansion, complete information can be retained when the object is rotated.

Ultimately, both unenhanced instances and enhanced instances collectively form the instances used for rapid dataset generation.

**2) Capture of background images**

Although the background images of the conveyor belt remain fixed during data collection, the color, surface texture, surface contamination, and brightness of the conveyor belt will change under different production environments. In order to rapidly create new datasets for new scenes, it is necessary to paste the instances onto backgrounds that represent different working conditions. Therefore, we have collected different background images with different brightness levels, colors, and surface contaminants to generate different datasets.

**3) Generate distribution points**

In actual solid waste stacking conditions, there are usually 2 to 3 objects stacked together, and more complicated situations rarely occur. To simulate a more realistic distribution of stacked objects, the point distribution method was proposed (**Fig 4(B)**). Firstly, the image is evenly divided into *n*^*2*^(*n* = *3*,*4*,*5*) regions, and the center point of each region is selected as a reference point. Then, the decision was made on whether each point should be considered as a paste point (red point), with a probability of 50% for each point. Finally, each point was subjected to a small random displacement to present a more complex distribution scenario.

**4) Paste instances onto the background images**

When the distribution points are generated, instances can be pasted into different background images according to the location of the distribution points. This paper uses the matrix operations capable of parallel computation to paste instances, which can accelerate the generation process of many images, as shown in **Table 1**.

**Table 1:** Method using matrix operations to paste instances.

**Input:** The mask image *mask*_*10*_ and *mask*_*01*_. The two-dimensional matrix of the pasted color instance image *crop*. Coordinates of distribution points generated in the background image (*m*_*p*_,*n*_*p*_). The two-dimensional matrix of the background image *BG*. **Output:** The background image *BG* after pasting instances.
**Pseudo-code:** *h = crop*.*shape[0] # h is the height of the instance image* *w = crop.shape[1] # w is the width of the instance image* *crop*_*10*_ = crop * mask_*10*_ BG = BG * mask_*01*_ *BG [n*_*p*_:*n*_*p*_*+h*, *m*_*p*_:*m*_*p*_*+w] = BG [n*_*p*_:*n*_*p*_*+h*, *m*_*p*_:*m*_*p*_*+w] + crop*_*10*_*[*:*h*,:*w]*

The specific implementation steps of this method are as follows:

- First, it is necessary to set the pixels inside the contour area to 1 and the pixel outside the contour point to 0 to obtain the mask image *mask*_*10*_, and *mask*_*01*_ is the negative of *mask*_*10*_.
- Multiply the matrix of the instance image and the mask image *mask*_*10*_ to get the instance image with background pixel 0.
- Multiply the matrix of the background image and mask image *mask*_*01*_ to get the background image with pixel 0 in the region to be pasted.
- Add the two-dimensional matrix of the instance image obtained in Step 2 and the two-dimensional matrix of the background image obtained in Step 3. Then the instance will be pasted into the background image.

Finally, the augmented dataset is obtained, and **Fig 4(C)** is the actual expansion result. It can be seen from steps 1 to 5 that when using the rapid generation method, many options can be chosen and can generate a rich and diverse dataset.

### Postprocessing of labels for synthetic stacked dataset

**Fig 6:**
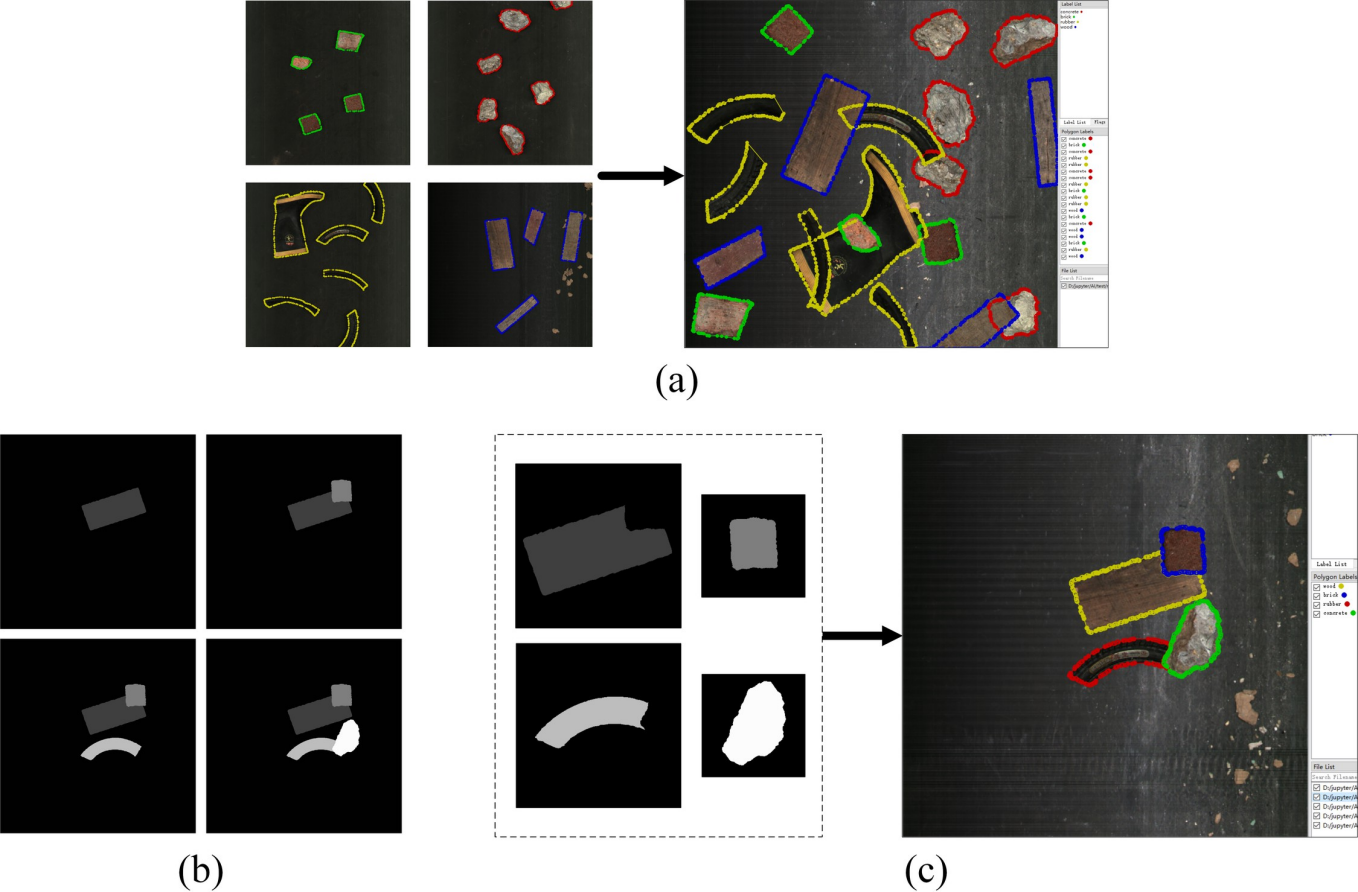
The label generation of rapid-generation datasets: (a) the generated contour point sets interfere with each other, (b) the pasting process of the instance grayscale image on the background grayscale image, (c) generate contour point sets from the grayscale images.

A large number of datasets of stacked waste can be generated through the method above, and instances in these images have sparse and dense distribution. In densely distributed images, mutual occlusion will occur between instances, changing the original contour. However, the label corresponding to the image is copied from the dataset before pasting and is fixed. If these labels are not processed, they will intersect and will not reflect the accurate contours of the instances in the generated image, as shown in **Fig 6(A)**.

In order to solve the problem of the labels interfering with each other, this section regenerates the contour point set by setting different gray values for different instances. Specific steps are as follows:

1. For each background image, the gray value of each pixel point is set to 1 to obtain the corresponding grayscale image *gray*_*BG*_. Then for each instance image, the gray value outside the contour is set to 0, and the gray value inside the contour is set to *num*_*i*_(*i* = *1*,*2*,…,*n*), in which *n* is the total number of instances in each generated image. The value range of *num*_*i*_ is shown in **Eq (5)**, and the corresponding instance grayscale image *gray*_*i*_ is obtained.
$$1<\;num{\;}_{1}<\;num{\;}_{2},\ldots ,<\;num{\;}_{n}<255$$ (5)
2. According to the generated distribution points, *gray*_*i*_ is successively pasted into the background grayscale image *gray*_*BG*_. The pasting method is the matrix operations pasted algorithm described above, as shown in **Fig 6(B)**. If each instance has mutual occlusion, the gray value *num*_*i*_ of the blocked pixel will also be covered.
3. According to the gray value of instance *num*_*i*_, the corresponding instance grayscale image $gray_{i}^{\prime }$ is extracted. Then the corresponding contour point set is generated through contour tracking, as shown in **Fig 6(C)**.

### Automatic labeling of real working condition dataset

Compared to synthetic dataset generated by rapid generation method, the captured real stacked dataset has more realistic stacking morphology and shadow effects. Additionally, the unlabeled real stacked datasets are more abundant and diverse in complex scenarios, allowing for on-the-fly collection during sorting. Moreover, numerous studies have demonstrated the effectiveness of incorporating real data into synthetic datasets. Therefore, we proposed two real stacked dataset annotation methods: an automatic labeling method based on semi-supervised self-training and an automatic labeling method based on RGB-D fusion edge detection.

### Automatic labeling method based on semi-supervised self-training

**Fig 7:**
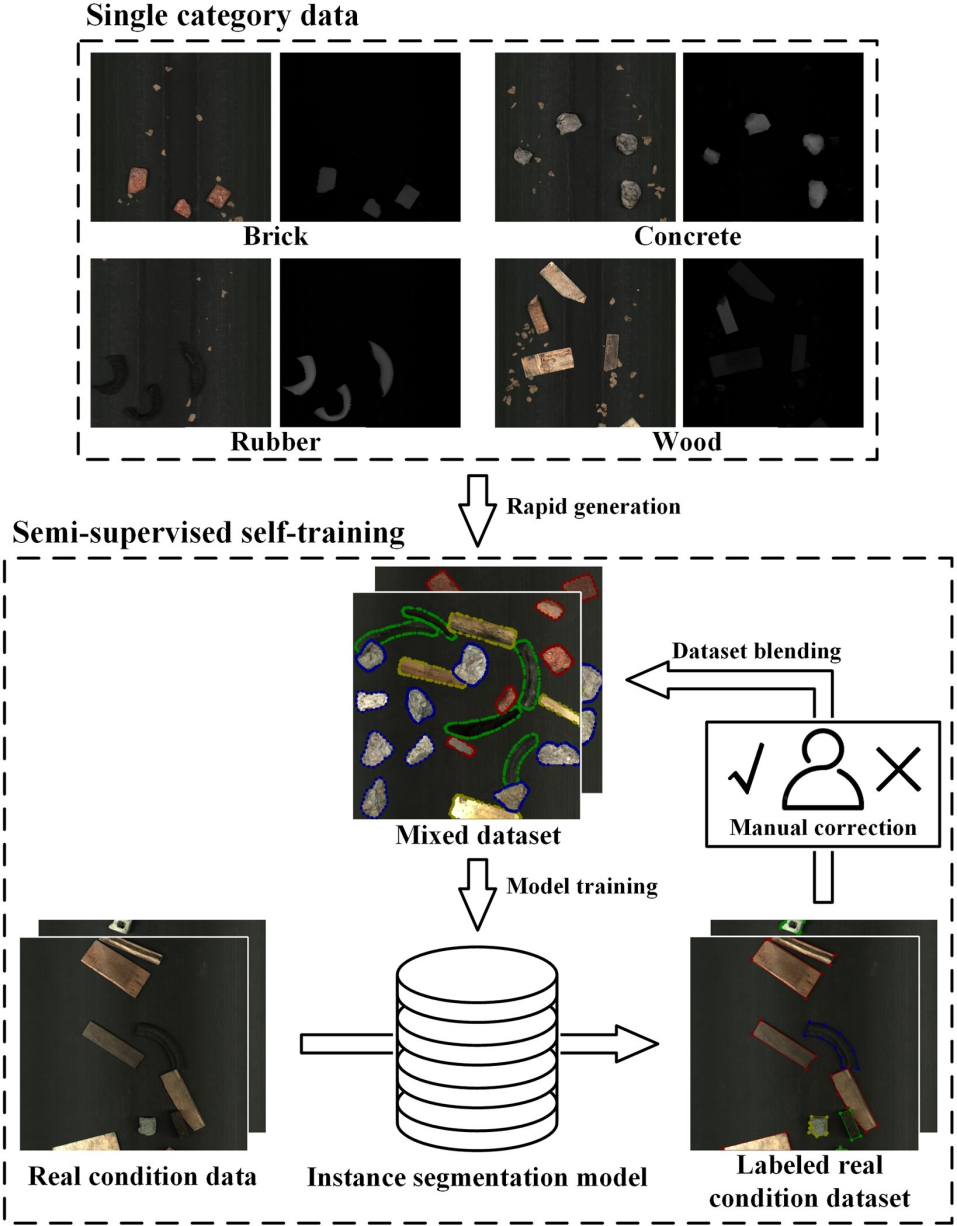
Semi-supervised self-training method based on rapid-generation datasets.

In order to reduce manual labeling, this paper proposes a semi-supervised learning method based on self-training [14] and the stacked dataset automatically generated in 2.4. The specific steps are shown in **Fig 7**.

Firstly, a stacked dataset is rapidly generated using a single-category dataset, and an instance segmentation model is trained based on this dataset. Then, the model trained with the rapid-generation dataset is utilized to predict real condition data. The predicted results are then converted into annotated contours and labels, thereby achieving automatic annotation. The labels generated by this method have a certain error rate, thus requiring manual correction. After the correction, the rapid-generation dataset and the real condition dataset are merged to create a mixed dataset to retrain the model. Continuously expanding the mixed dataset and retraining the model can accumulate more data and improve the prediction model’s accuracy.

Compared to manual annotation methods, the proposed approach saves time and effort as it does not require manual annotation, only correction. In contrast to solely using rapid-generation datasets, incorporating datasets under real working conditions is more beneficial for the model to adapt to real working conditions. Additionally, it can provide continuous annotated datasets while the equipment is in production.

### Automatic labeling method based on RGB-D fusion edge detection

However, self-training methods are prone to mislabeling because rapid-generation datasets are challenging to simulate natural shadows, heights, and edges. For traditional image algorithms, stacked objects and polluted backgrounds can cause serious interference with detection using only RGB or depth images.

**Fig 8:**
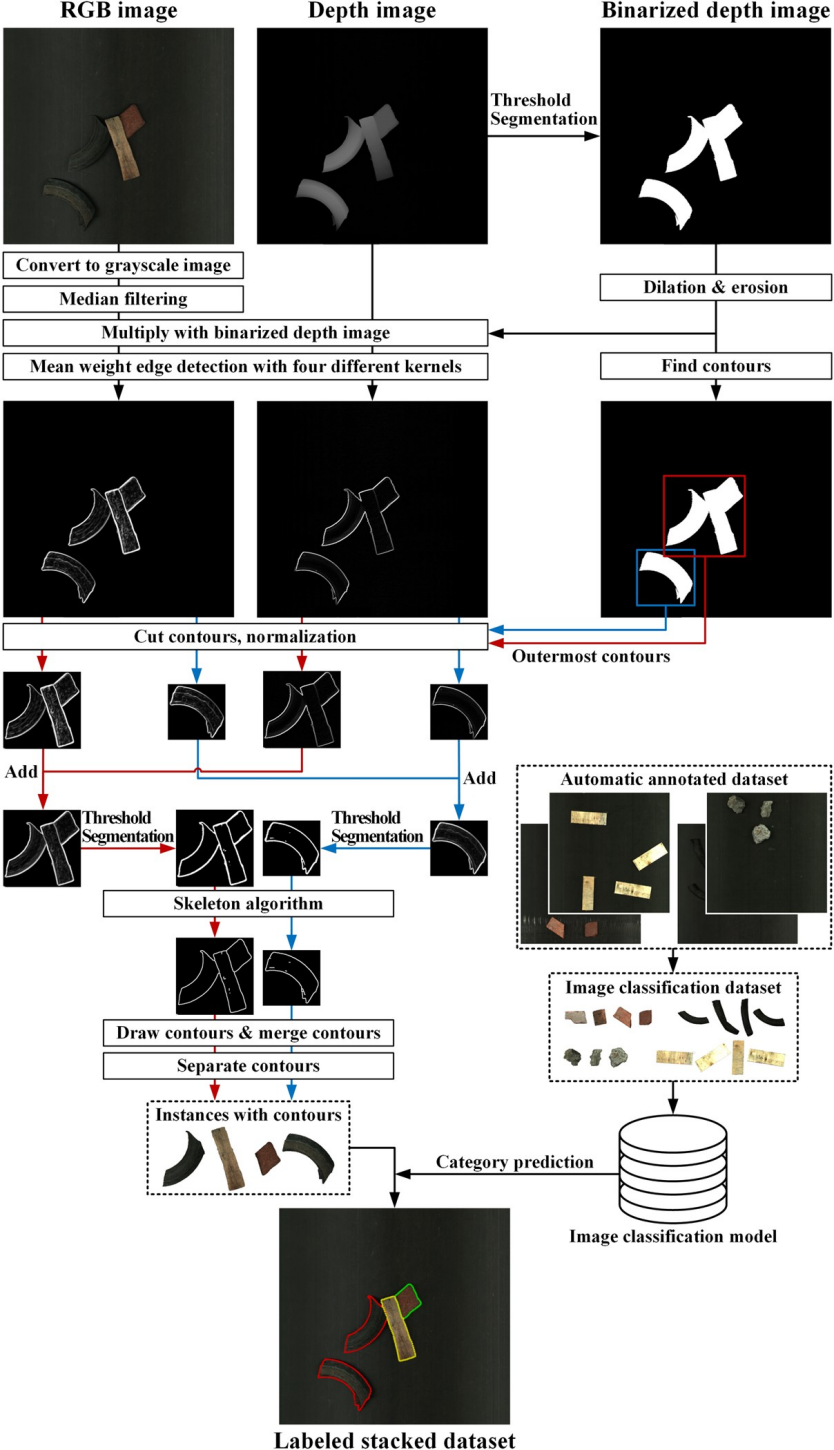
Automatic labeling method of real stacked datasets based on RGB-D fusion edge detection.

Therefore, the RGB-D fusion edge detection method is proposed specifically for fully automatic annotation of real stacked datasets. It combines RGB-D edge detection with rapid-generation dataset to automatically generate real stacked dataset. The specific steps are shown below (**Fig 8**).

1. Firstly, the depth image *depth*_*src*_ is segmented with a threshold value of 20 (maxval = 255). Then the image is adjusted with dilate and erode operations (kernel = (3,3), iterations = 2), to get the threshold segmentation depth image *depth*_*thresh*_. Dilate fills white pixels around with white parts, achieving the effect of contour expansion, while erode is the opposite. Finally, the contour search method [26] is used to get the outer contours of all stacked parts.
2. The RGB image *rgb*_*src*_ is converted to grayscale image *rgb*_*gray*_, and the median filter is used to remove surface texture interference (ksize = 9) to get *rgb*_*blur*_. After filtering, *depth*_*src*_ and *rgb*_*blur*_ are multiply with *depth*_*thresh*_ respectively, to filter out background interference. Following that, four 7×7 convolution kernels with lower center weights: *X*,*X*^*T*^,*Y*,*Y*^*T*^ (**Eq (6)**) are designed to conduct edge detection on the processed RGB and depth image to obtain the edge detected image *rgb*_*edge*_ and *depth*_*edge*_. The designed kernels can allow the algorithm focus on more prominent edge differences.
$$X=\left[\begin{array}{ccccccc} 1 & 1 & 1 & 0 & 0 & 0 & 0\\ 1 & 1 & 1 & 1 & 0 & 0 & 0\\ 1 & 1 & 1 & 0 & 0 & 0 & 0\\ 0 & 1 & 0 & 0 & 0 & -1 & 0\\ 0 & 0 & 0 & 0 & -1 & -1 & -1\\ 0 & 0 & 0 & -1 & -1 & -1 & -1\\ 0 & 0 & 0 & 0 & -1 & -1 & -1 \end{array}\right],Y=\left[\begin{array}{ccccccc} 0 & 0 & 1 & 1 & 1 & 0 & 0\\ 0 & 1 & 1 & 1 & 1 & 1 & 0\\ 0 & 0 & 1 & 1 & 1 & 0 & 0\\ 0 & 0 & 0 & 0 & 0 & 0 & 0\\ 0 & 0 & -1 & -1 & -1 & 0 & 0\\ 0 & -1 & -1 & -1 & -1 & -1 & 0\\ 0 & 0 & -1 & -1 & -1 & 0 & 0 \end{array}\right]$$ (6)
3. The *rgb*_*edge*_ and *depth*_*edge*_ are cropped according to the outermost contours obtained by *depth*_*thresh*_, then each of the cropped images are normalized. Next, the corresponding cropped images are added with the same weight, and then the added images are segmented with a fixed threshold to highlight the actual contours.
4. The skeletonize operator [27] is used to simplify the irregular contours. Then the burr contours, contours with no area, and contours encircled by other contours are removed. Meanwhile, contours with small area (less than 20% of the maximum area) are merged into the closest contour with the slightest difference between the average R, G, and B values (calculation formula). Up to this point, instances with labeled contours are obtained.
5. In the last step, the image classification model trained by the automatically labeled dataset in section "Automatic annotation of instances" is used to recognize the category of instances obtained from step **4)**, and finally a real working condition dataset with labels and contours is obtained.

Both automatically annotated real stacked datasets are manually corrected to avoid labeling errors.

## Experiment

To validate the effectiveness of the proposed method in this paper, the aforementioned approach was utilized to generate datasets, which were then divided into training and testing sets. The Mask R-CNN instance segmentation algorithm was trained using the generated datasets. Subsequently, the algorithm was utilized to predict the test set, and the performance was evaluated using three metrics: precision, recall, and F1-score.

### Architecture of detection network

**Fig 9:**
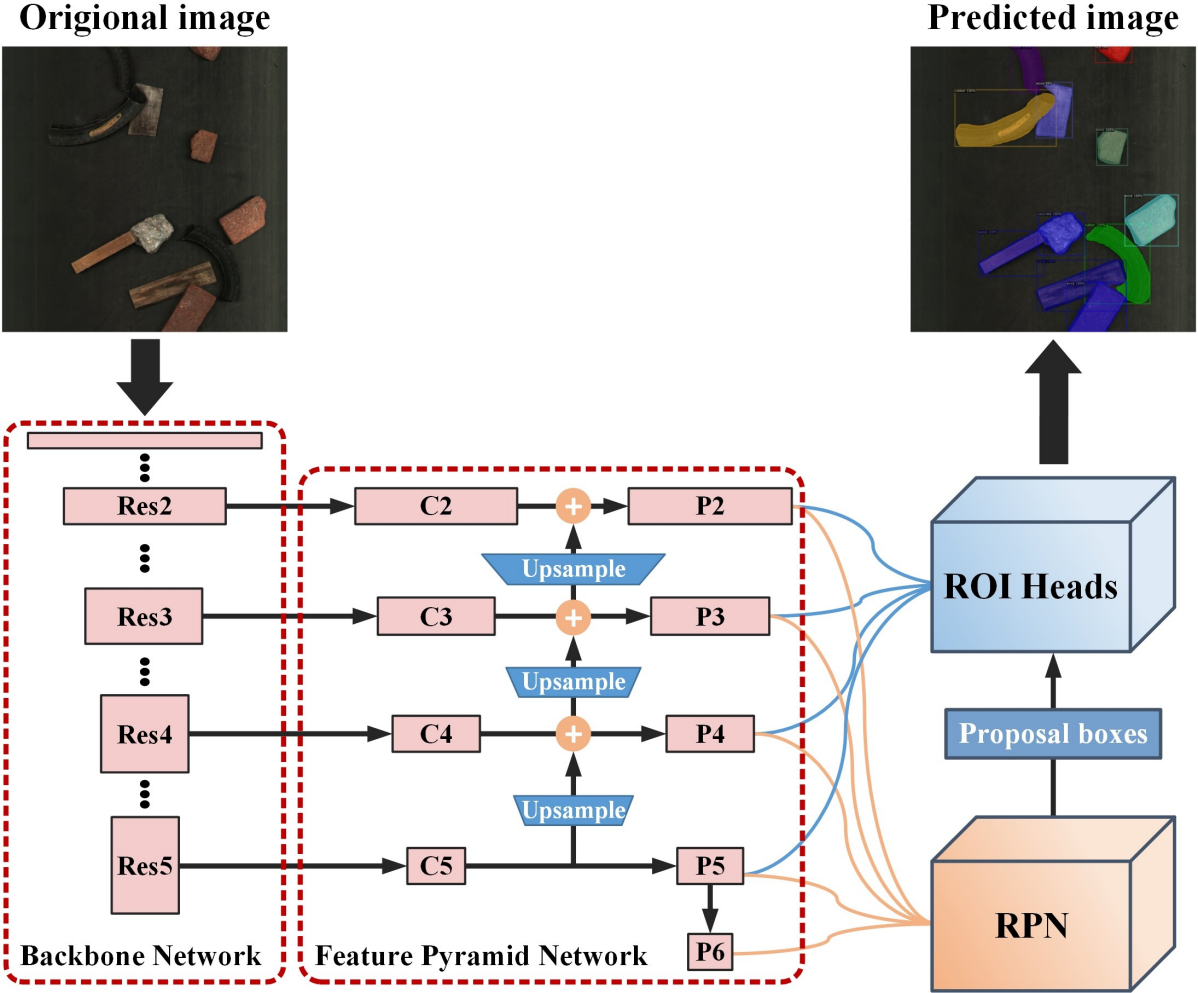
Structure of the instance segmentation model.

The rapid generation method proposed in this article is adaptable and can be used on different object detection algorithms. In order to verify the effectiveness of this method, this paper chose the classic instance segmentation algorithm Mask R-CNN [28] as the experimental model with ResNet101 [29] as the model’s backbone network and feature pyramid network (FPN) for multi-scale feature fusion [30]. The structure of the detection algorithm is shown in the **Fig 9**.

### Implementation details

All experiments followed the same training methodology, and were conducted on a deep learning workstation equipped with a i9 9900k Central Processing Unit (Intel, Amercia) and two 2080Ti Graphics Processing Units (NVIDIA, America). The operating system of the workstation is based on Windows 10. The programming language used is Python, and the instance segmentation algorithm is implemented using the TensorFlow 1.15 framework. To accelerate the model training process, this paper adopted the pre-trained weight trained on the COCO dataset to initialize the weight of our model. The learning rate is set to 0.001, the batch size is 4, and the image size of the model input is 512*512.

### Datasets

This paper collected datasets under two working conditions, the simple working condition (A) and the complicated working condition (B). Under simple working conditions, the distribution of objects is scattered, and the conveyor belt is clean. While in the complicated working condition, solid waste stacking is standard, and the conveyor belt is covered with unrelated interferences like waste paper and gravel (**Fig 1**). The collected datasets are shown in **Table 2**.

**Table 2:** The experimental datasets.

	Clean condition	Number of images	Interference condition	Number of images
Training set	A_auto_	556	B_auto_	302
	A_CP_	2386	B_CP_	1130
	A_manual_	1834	-	-
Test set	A_test_	777	B_test_	336

**Training set:** Under the simple working condition, 626 images were collected as the training set A_auto_; under conditions with interferents, 302 images were collected as the training set B_auto_. Objects of the same category are collected together and sparsely placed in each image and generated labels with the method in section "Automatic annotation of instances". In order to verify the validity of the rapid generation method, the training set A_auto_ was used to generate 2386 images as the training set A_CP_, and the training set B_auto_ was used to generate 1130 images as the training set B_CP_. For comparison, 1884 real images were captured and labeled manually as the training set A_manual_. It should be noted that the number of images generated by the rapid generation method depends on the number of instances, the number of grid points, and the enhancement methods used. In this study, the size of the A_CP_ is suitably designed, but the number of images is still slightly more than A_manual_. This could potentially introduce bias, and improving the accuracy of generating image quantities will be a further area for enhancement.

**Test set:** To show the effectiveness of the designed method, 777 images were collected as the test set A_test_ under the simple condition, and 336 images were collected as the test set B_test_ under the complicated condition. All images in the test set were manually annotated to measure the model’s performance.

### Evaluation metrics

Accuracy is a general index, and the prediction result of each sample will affect the accuracy. There will be category imbalance in multi-classification models, and it is impossible to evaluate the model’s recognition of each category using accuracy. Therefore, metrics derived from the confusion matrix. The confusion matrix can calculate the precision and recall of each category. As shown in **Eqs (7)** and **(8)**: TP stands for True Positive, which means the sample is positive and correctly identified. FP stands for False Positive, representing the sample as negative but wrongly identified as positive. FN is False Negative, meaning the sample is positive but wrongly identified as negative.


$$\;Precision\;=\frac{TP}{TP+FP}$$ (7)

$$Recall\;=\frac{TP}{TP+FN}$$ (8)

Precision represents the proportion of correct predictions that account for all optimistic predictions. Recall represents the proportion of correct predictions that account for all actual positives. Precision and Recall will affect each other. When one becomes higher, the other becomes lower. To balance the influence, F1-score is introduced to comprehensively evaluate the multi-classification model, as shown in **Eq (9)**.


$$F_{1}=\frac{2\;Precision\;\;Recall}{\;Precision+Recall}$$ (9)

## Results and discussion

This section presents the experimental results and analysis of the study. Firstly, it compares the annotation performance between manually annotated datasets and automatically generated datasets. Then, it presents the instance segmentation performance under simple and complex conditions, followed by analyses of the results based on the principles of the detection algorithm and the dataset.

### The comparison of annotation

**Fig 10:**
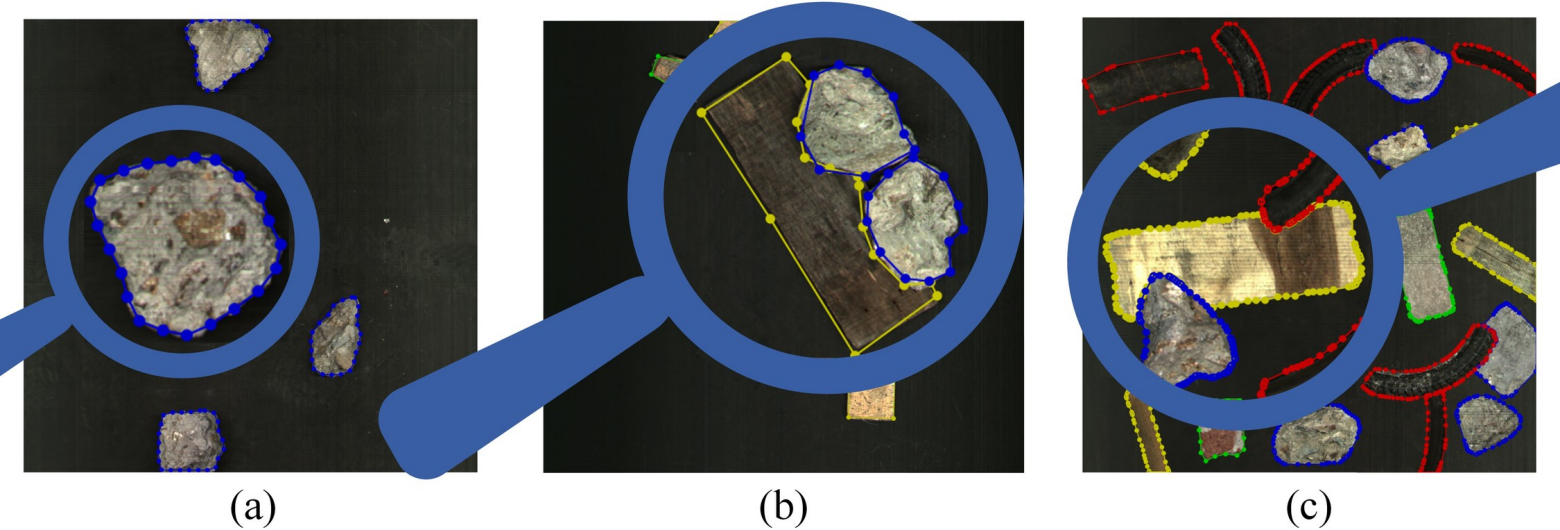
Comparison of annotations with different methods: (a) automatically labeled data, (b) manually labeled data, (c) labeled data generated by rapid generation.

**Fig 10(B)** is the data labeled by authors. When encountering irregular objects, it requires a significant number of points to annotate them accurately, which in turn takes more time. Moreover, there are numerous stacked objects in field working conditions. It is even more difficult to use manual labeling. **Fig 10(A)** is the labeled data automatically generated by combining RGB and depth images. As seen from the **Fig**ure, contours in the automatically labeled data fit objects well, even if they are irregular. **Fig 10(C)** depicts the labeled data generated by the proposed method used to simulate the field working conditions. Compared to **Fig 10(B)**, the quality of contours generated with our approach is very high, even if objects are adhered to and stacked to each other.

In the experiment, it took around 48 hours for authors to label the dataset A_manual_.

On the other hand, labels for dataset A_auto_/B_auto_ and dataset A_CP_/B_CP_ could be automatically generated within 5 minutes. By utilizing this approach, a significant amount of manpower and time can be saved, while obtaining more accurate contour annotations, which is beneficial for rapidly improving the quality of the detection model.

### Experimental comparison under the simple working condition

**Fig 11:**
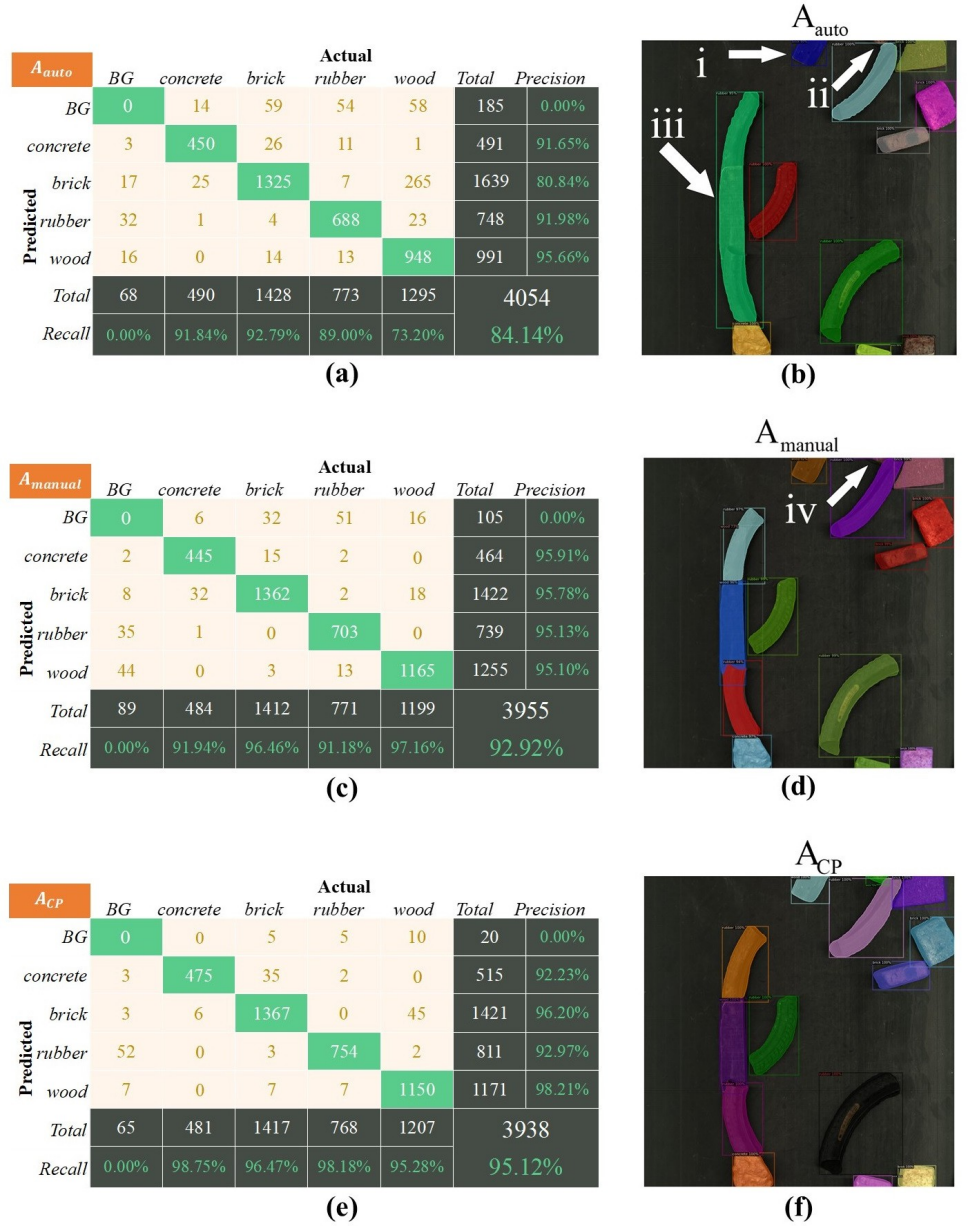
Performance of trained models on A_test_, the white arrows indicate areas where the model’s predictions are wrong: (a), (c), (e) is the confusion matrices on A_test_, (b), (d), (f) is the predicted image results of A_test_.

The test set used in the simple condition was A_test_. The training set used in this experiment included the automatically labeled dataset A_auto_, the rapid-generation dataset A_CP_, and the manually labeled dataset A_manual_. Moreover, all the models trained by the above training set were tested on A_test_. The experimental results are shown in **Fig 11** and **Table 3**, and BG denotes the background, which represents the object that was not detected.

**Table 3:** F1-Scores of all trained models on the test set.

Test set	Training set	F1_concrete_	F1_brick_	F1_rubber_	F1_wood_	F1_average_
A_test_	A_auto_	91.74	86.40	90.47	82.94	87.89
	A_manual_	93.88	96.12	93.11	96.12	94.81
	A_CP_	**95.38**	**96.33**	**95.50**	**96.72**	**95.98**
B_test_	A_manual_	93.38	61.44	97.08	92.00	85.97
	B_CP_	**98.37**	**97.17**	**96.56**	**98.87**	**97.74**

1. Analysis of experimental results of the training set A_auto_.
  All objects in training set A_auto_ were sparsely placed. However, the test set A_test_ under the simple condition contained many densely distributed objects, so the model trained by A_auto_ had a poor effect in detecting the conglutinated and stacked objects. The prediction results of this model on A_test_ are shown in **Fig 11(B)**, where the white arrow points to the error of model detection:
  - The model mistakenly identified a piece of wood at the edge of the image as a brick, which is likely due to the scarcity of boundary cases in the dataset;
  - The model missed recognition of a tiny object at the edge of the image. That is, the model’s recognition ability of tiny objects was not good enough, and it was easy to mistake them for background and not detect them;
  - The model mistakenly identifies three instances connected as one instance, meaning that the model is not good enough to recognize the touched and stacked instances.
  As can be seen from the confusion matrix in **Fig 11(A)**, the detection effect of this model for different categories was not stable enough. The precision of the wood in the model can reach 95.66%, while the precision for brick was only 80.84%. Moreover, the recall for wood was only 73.20%. As can be seen from the confusion matrix, the model identified the wood as bricks easily.
2. Analysis of experimental results of the training set A_manual_.
  Images in A_manual_ were collected in field working conditions, so the distribution of objects in A_manual_ was the closer to the test set. The model trained on A_manual_ exhibits a high level of detection accuracy. It can be seen from **Fig 11(A)** that the model also performed well in recognition of the conglutinated and stacked objects. However, it can be seen from the error at the **(iv)** arrow that the model should be improved to recognize the tiny object at the edge of the image, and it also recognized it as the background.
  It can be seen from the confusion matrix in **Fig 11(C)** that the precision of the model for all categories exceeded 95%, but there were still some cases where objects and backgrounds were confused.
3. Analysis of experimental results of the training set A_CP_.
  Images and labels in training set A_CP_ were extended based on A_auto_. The images in A_CP_ had a similar distribution to the dataset of actual working conditions, including sparse and dense distribution. The model trained by A_CP_ had the best overall performance in test set A_test_, which can distinguish the conglutinated and stacked objects and detect tiny objects, as shown in **Fig 11(F)**.
  It can be seen from the confusion matrix in **Fig 11(E)** that the Recall of the model trained by A_CP_ was over 95%, and the cases of objects and backgrounds confusion were much less than those of the above two training sets.

**Table 3** shows the F1-scores of all trained models on test set A_test_. In all categories of F1-scores in **Table 3**, A_CP_ scored the highest, followed by A_manual_, and A_auto_ scored the lowest. Furthermore, the average F1-score of the model trained by A_CP_ on A_test_ reached 95.98, which is higher than the model trained on A_manual_.

In the experiment, although the model performance was poorer with the automatically generated dataset, the model performed well after the dataset was augmented with the fast generation method. The fast generation dataset even outperformed the manually labeled dataset. Therefore, the rapid generation method proposed can generate a huge dataset to simulate complex scenarios, compensating for the drawback of generating less realistic images and achieving better results than manually labeled dataset.

### Experimental comparison under the complicated working condition

In the practical application, sorting conditions may change, resulting in the degradation of the detection performance of models. Therefore, this experiment simulated the change in actual working conditions by increasing the distribution density of objects and adding interferences on the conveyor belt.

**Fig 12:**
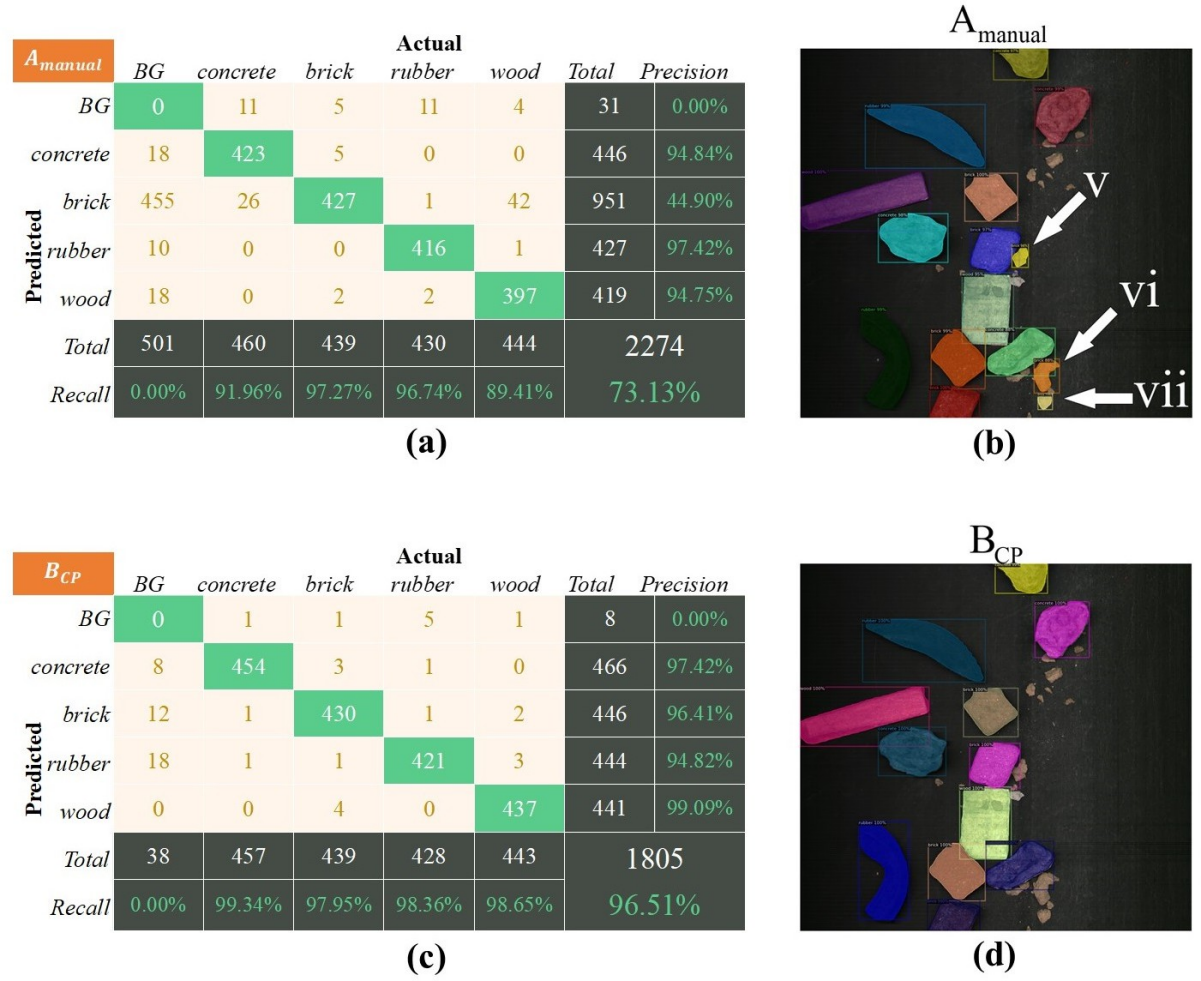
Performance of trained models on B_test_: (a), (c) is the confusion matrices on B_test_, (b), (d) is the predicted image results of B_test_.

The test set used in this experiment was B_test_. The training sets included the manually annotated dataset A_manual_ and the rapid-generation dataset B_CP_. All the models trained by the above training set were tested on test set B_test_. The experimental results obtained by the test were shown in **Fig 12** and **Table 3**.

1. Analysis of experimental results of the training set A_manual_.
  As seen from the above section, the manual training set A_manual_ significantly improved the model under the simple working condition. However, A_manual_ performed poorly when the working condition changed. This is because A_manual_ (except for the captions) As shown in **Fig 12(B)**, although the model can classify the construction solid waste well, the model misidentified the three interferers as bricks, as shown in the area pointed to by (v), (vi), and (vii). From the confusion matrix in **Fig 12(A)**, it can be observed that the model misclassified 455 interferences and background regions as bricks, resulting in an accuracy of only 44.90% for bricks.
2. Analysis of experimental results of the training set B_CP_.
  When the operational condition changes, the B_CP_ can be generated quickly using the proposed rapid generation method. The model trained with B_CP_ demonstrates excellent performance on the test set B_test_. This is because the rapid-generation dataset includes new backgrounds, distractions, lighting conditions, and other information. When instances are pasted onto new backgrounds, it enables the model to adapt to new scenarios. As shown in **Fig 12(D)**, the model accurately identified all the construction solid waste and did not misidentify the interferers. As can be seen in **Fig 12(C),** the Recall of all categories was over 97%, and the lowest precision reached 94.82%.

**Table 3** shows the F1-scores of all the trained models tested on test set B_test_. It can be seen that the F1-scores of the training set B_CP_ were significantly higher than A_manual_. Finally, the average F1-score of B_CP_ reached 97.74.

When faced with a working condition that needs detection, the traditional method is to obtain a high-quality dataset through time-consuming and laborious manual annotation. However, in actual applications, the working conditions could be Changeable. It can be seen from this experiment that when faced with new conditions, the initially labeled dataset cannot make the model achieve good performance.

The proposed method in this study enables rapid generation of datasets under complex working conditions without the need for manual annotation. Additionally, the models trained on rapid-generation dataset exhibit excellent performance and can be continuously improved through dataset generation. Therefore, the dataset generation method presented in this paper allows for the quick deployment of models in various working conditions while ensuring high recognition accuracy and continuous optimization.

## Conclusions

This paper proposes a rapid dataset generation method to produce stacked construction solid waste datasets without manual labeling. At the same time, two additional automatic annotation methods for real condition datasets were proposed. Firstly, an acquisition and detection platform was built to automatically collect RGB-D images and instances. Then the stacked construction solid waste dataset was generated based on the designed distribution points generation theory and data augmentation algorithm, which facilitates the swift and automated data collection and the generation of datasets, thereby saving time and workload. Moreover, two automatic labeling methods based on semi-supervised learning and edge detection were proposed, which can quickly annotate real stacked datasets without manual annotation. This enables rapid expansion of high-quality datasets, allowing continuous data annotation during waste sorting to enhance model performance. Finally, two working conditions were designed to verify the method’s effectiveness. Under the simple working condition, the F1-score of the dataset generated by the rapid generation method was 95.98, which is more than 94.81 for the manually labeled dataset. When the working condition was changed to the complicated condition, the F1-score of the dataset obtained by manual annotation was only 85.97. However, the rapid generation method can get the new training set under the complicated working condition within a brief time. Also, the F1-score of the new training set under the second working condition reached 97.74. The results demonstrate the effectiveness of the rapid-generation and automatic annotated dataset.

From an engineering application perspective, the methods proposed in this paper can save a significant amount of annotation time and effort. They also enable the quick creation of new datasets to adapt to changing working conditions. However, there are still some limitations in this study. **1)** The amount and variety of samples in the paper are limited, and the complexity of real working conditions is not fully represented. Further research will be conducted in construction sites to obtain more complex data and working conditions. **2)** The proposed method for data generation and automatic annotation may not fully handle extreme situations, such as multiple objects stacked together. More robust methods are still needed. **3)** Although the automated dataset generation and annotation greatly reduce manual costs, human verification is still difficult to avoid.
